# Nitric oxide donors increase PVR/CD155 DNAM-1 ligand expression in multiple myeloma cells: role of DNA damage response activation

**DOI:** 10.1186/s12885-015-1023-5

**Published:** 2015-01-22

**Authors:** Cinzia Fionda, Maria Pia Abruzzese, Alessandra Zingoni, Alessandra Soriani, Biancamaria Ricci, Rosa Molfetta, Rossella Paolini, Angela Santoni, Marco Cippitelli

**Affiliations:** 1 Department of Molecular Medicine, Istituto Pasteur-Fondazione Cenci Bolognetti, Sapienza University of Rome, Viale Regina Elena 291, 00161 Rome, Italy; 2 Istituto Mediterraneo di Neuroscienze Neuromed, Pozzilli, IS Italy

**Keywords:** Multiple myeloma, Nitric oxide, DNAM-1, Natural killer, DNA damage response, Chemoimmunotherapy

## Abstract

**Background:**

DNAX accessory molecule-1 (DNAM-1) is an activating receptor constitutively expressed by macrophages/dendritic cells and by T lymphocytes and Natural Killer (NK) cells, having an important role in anticancer responses; in this regard, combination therapies able to enhance the expression of DNAM-1 ligands on tumor cells are of therapeutic interest. In this study, we investigated the effect of different nitric oxide (NO) donors on the expression of the DNAM-1 ligand Poliovirus Receptor/CD155 (PVR/CD155) in multiple myeloma (MM) cells.

**Methods:**

Six MM cell lines, SKO-007(J3), U266, OPM-2, RPMI-8226, ARK and LP1 were used to investigate the activity of different nitric oxide donors [DETA-NO and the NO-releasing prodrugs NCX4040 (NO-aspirin) and JS-K] on the expression of PVR/CD155, using Flow Cytometry and Real-Time PCR. Western-blot and specific inhibitors were employed to investigate the role of soluble guanylyl cyclase/cGMP and activation of the DNA damage response (DDR).

**Results:**

Our results indicate that increased levels of nitric oxide can upregulate PVR/CD155 cell surface and mRNA expression in MM cells; in addition, exposure to nitric oxide donors renders myeloma cells more efficient to activate NK cell degranulation and enhances their ability to trigger NK cell-mediated cytotoxicity. We found that activation of the soluble guanylyl cyclase and increased cGMP concentrations by nitric oxide is not involved in the up-regulation of ligand expression. On the contrary, treatment of MM cells with nitric oxide donors correlated with the activation of a DNA damage response pathway and inhibition of the ATM /ATR/Chk1/2 kinase activities by specific inhibitors significantly abrogates up-regulation.

**Conclusions:**

The present study provides evidence that regulation of the PVR/CD155 DNAM-1 ligand expression by nitric oxide may represent an additional immune-mediated mechanism and supports the anti-myeloma activity of nitric oxide donors.

**Electronic supplementary material:**

The online version of this article (doi:10.1186/s12885-015-1023-5) contains supplementary material, which is available to authorized users.

## Background

Multiple myeloma (MM) is a deadly hematologic cancer characterized by latent accumulation of clonal secretory plasma cells in the bone marrow. Despite advances in therapeutic strategies, MM remains an incurable disease with a median survival around 4–5 years in adults [1]. However, in the past decade, the use of autologous hematopoietic stem cell transplantation (HSCT) and the introduction of new drugs, such as bortezomib and IMiDs, have improved survival [2-5].

Increasing evidence in myeloma patients has shown that Natural Killer (NK) cells can elicit potent allogeneic and autologous responses to myeloma cells, strongly supporting their anti-tumor potential in response to immunomodulatory drugs or following allogeneic stem cell transplantation [6-8]. In this regard, several studies have shown that triggering of different activating receptors, such as DNAX accessory molecule-1 (DNAM-1), NK group 2D (NKG2D) and Natural Cytotoxicity Receptors (NCRs), is involved in the recognition and killing of MM cells by NK cells [9-11]; moreover, MM cells can express the DNAM1-ligands (DNAM1Ls) PVR/CD155 and Nectin-2 (Nec-2) [12] and the NKG2D-ligands (NKG2DLs) MICA/B and ULBPs on the cell surface [9,12,13].

Nitric oxide (NO) is a reactive radical, highly diffusible pleiotropic regulator of many different biological pathways, including vasodilatation, neurotransmission and macrophage-mediated responses to infections. It is generated from molecular oxygen and the amino acid _L_-arginine through the action of the nitric oxide synthase (NOS) enzymes; three isoforms of NOS have been identified, a neuronal form (nNOS/NOS1) and endothelial form (eNOS/NOS3) which are both constitutively expressed enzymes producing physiological levels of NO, and an inducible form (iNOS/NOS2) which produces high levels of NO in a sustained manner [14-16]. In the last years, the relationship between NO and the pathology of malignant disorders has been the subject of numerous studies; although the three NOS isoforms are known to be present in most tumors and generally expressed at higher levels compared to their normal tissue counterparts, their functional role still remains incompletely elucidated [17,18]. In this regard, a concentration-dependent dual nature of NO has been revealed, where low concentrations of NO can promote invasion and metastases in different tumor models or, on the contrary, high NO levels (e.g. immune cell-generated NO) and the different reactive nitrogen species (RNS) produced can inhibit tumor growth and metastases (reviewed in [17,19,20]). Thus, NO may play different roles in regulating cancer microenvironment and progression, which can be cell-type and context specific.

These observations suggest that tumor immune rejection through NO-dependent mechanism(s) can represent an interesting promise for future tailored immunotherapeutic anticancer strategies.

Our laboratory has recently shown that suboptimal doses of different drugs, such as genotoxic chemotherapeutics, inhibitors of the HSP-90 protein or of the GSK3 kinase, can increase the expression of several NK activating ligands on MM cells, via induction of specific regulatory transcriptional pathways [12,21,22]; the up-regulation of these ligands on MM cells is associated with their ability to trigger increased NK cell degranulation. At this regard, expression of DNAM-1 ligands and in particular PVR/CD155 can be regulated by activation of a DNA damage response (DDR) pathway induced by anticancer drugs (e.g. doxorubicin or melphalan) or, in a different context, by monocyte-derived reactive oxygen species (ROS) in Ag-induced T cell proliferation [23].

Here, we analyzed the possibility that treatment of MM cells with different NO-donors could regulate the expression of the NK cell activating ligand PVR/CD155 and, in turn, modify NK cell recognition and cytotoxicity against these cancer cells.

Our results indicate that increased levels of NO can enhance surface expression of PVR/CD155 on MM cell lines, rendering these cells more susceptible to NK cell mediated killing via DNAM-1 recognition. We found that activation a DDR by NO is critical for these mechanisms since pharmacological inhibition of ATM/ATR or Chk1/2 kinases as well as knockdown of E2F1, a transcription factor activated in response to DNA damage, significantly reduced NO-induced upregulation of PVR/CD155.

Overall, our data demonstrate that NO can regulate DNAM-1 ligand expression on MM cells, suggesting novel roles of NO in immune response(s) to multiple myeloma.

## Methods

### Cell lines

The human MM cell lines SKO-007(J3), U266, OPM-2, ARK, RPMI-8226 and LP1 were kindly provided by Prof. P. Trivedi (Sapienza University of Rome, Italy). SKO-007(J3) cells transduced with a lentiviral vector expressing shRNAs targeting E2F1 have been already described [24]. The erythroleukemia cell line K562 and MM cell lines were maintained at 37°C and 5% CO2 in RPMI 1640 (Life Technologies, Gaithersburg, MD) supplemented with 10% FCS, 2 mM glutamine and 100 units/ml penicillin-streptomycin (complete medium). All cell lines were mycoplasma-free (EZ-PCR Mycoplasma Test Kit, Biological Industries).

### Reagents and antibodies

The nitric oxide donors DETA-NO [2,2′-(hydroxynitrosohydrazono) bis-ethanimine], NCX4040 (NO-aspirin), JS-K [O2-(2,4-Dinitrophenyl) 1-[(4-ethoxycarbonyl)piperazin-1-yl]diazen-1-ium-1,2-diolate], caffeine, LY294002 and the inhibitor of nitric oxide-sensitive guanylyl cyclase ODQ (1H-[1,2,4]Oxadiazolo[4,3-a]quinoxalin-1-one) and Bafilomycin A1, were purchased from Sigma-Aldrich (St. Louis, MO). The Chk1/2 pharmacologic inhibitors SB218078 and UCN-01 were purchased from Calbiochem, EMD Chemicals (Darmstadt, Germany). C12FDG was from Invitrogen (Frederick, MD). The nitric oxide donor DETA-NO (2 moles of NO**•** per mole of compound and a half-life of 20 h at 37°C), is ideal for the treatment of cells over long periods of time (e.g. 24–48 h). JS-K (an anti-cancer agent belonging to the diazeniumdiolate family of compounds), is designed to release nitric oxide (NO) in a sustained and controlled manner within a cell, when metabolized by glutathione *S*-transferases (GSTs).

The following monoclonal antibodies (mAbs) were used for immunostaining or as blocking Abs: anti-PVR/CD155 (SKII.4) kindly provided by Prof. M. Colonna (Washington University, St Louis, MO), anti-CD56 (C218) mAb was provided by Dr. A. Moretta (University of Genoa, Genoa, Italy), anti-DNAM-1 (DX11) from Serotec (Oxford, UK), anti-Nec-2 (R2.525) from BD Biosciences (San Jose, CA), anti-TIGIT (MBSA43) from eBioscience Inc. (San Diego, CA). APC Goat anti-mouse IgG (Poly4053), anti-CD3/APC (HIT3a), anti-CD56/PE (HCD56), mouse IgG1/FITC, /PE or /APC isotype control (MOPC-21) were purchased from BioLegend (San Diego, CA). Anti-CD107a/FITC (H4A3) was purchased from BD Biosciences (San Jose, CA).

### Immunofluorescence and flow cytometry

MM cell lines were cultured in 6-well tissue culture plates for 48 h at a concentration of 2 × 10^5^ cells/ml in the presence of different concentrations of drugs. The expression of PVR/CD155 on MM cells was analyzed by immunofluorescence staining using an anti-PVR/CD155 unconjugated mAb, followed by secondary GAM-APC. In all experiments, cells were stained with Propidium Iodide (PI) (1 μg/ml) in order to assess cell viability (always higher than 90% in the different treatments). Nonspecific fluorescence was assessed by using an isotype-matched irrelevant mAb (R&D System) followed by the same secondary antibody. Fluorescence was analyzed using a FACSCalibur flow cytometer (BD Bioscience, San Jose, CA) and FlowJo Flow Cytometric Data Analysis Software (Tree Star, Inc. Ashland, OR).

Intracellular NO• levels were measured by flow cytometry in cells loaded with the NO-sensitive dye DAF2-DA [4,5-Diaminofluorescein-diacetate (Molecular Probes, Invitrogen, San Diego, CA)]. Cells were gated by forward/side scatter and fluorescence was recorded on the FL-1 channel according to the manufacturer’s protocol.

### Degranulation assay

NK cell-mediated cytotoxicity was evaluated using the lysosomal marker CD107a as previously described [21]. As source of effector cells, we used primary NK cells obtained from PBMCs isolated from healthy donors by Lymphoprep (Nycomed, Oslo, Norway) gradient centrifugation and then co-cultured for 10 days with irradiated (30 Gy) Epstein-Barr virus (EBV)-transformed B-cell line RPMI 8866, without the addition of recombinant IL-2, at 37°C in a humidified 5% CO2 atmosphere as previously described [25]. Informed consent in accordance with the Declaration of Helsinki was obtained from all donors, and approval was obtained from the Ethics Committee of the Sapienza University of Rome, Italy. On day 10, the cell population was routinely more than 90% CD56^+^CD16^+^CD3^−^, as assessed by immunofluorescence and flow cytometry analysis. Drug-treated MM cells were washed twice in complete medium and then incubated with NK cells at the effector:target (E:T) ratio of 2.5:1, in a U-bottom 96-well tissue culture plate in complete medium at 37°C and 5% CO2 for 2 h. Thereafter, cells were washed with PBS and incubated with anti-CD107a/FITC (or cIgG/FITC) for 45 min at 4°C. Cells were then stained with anti-CD3/APC, anti-CD56/PE to gate the CD3^−^CD56^+^ NK cell population. In some experiments, cells were pre-treated for 20 min at room temperature with anti-DNAM-1 or anti-TIGIT blocking mAb. Fluorescence was analyzed using a FACSCalibur flow cytometer (BD Bioscience, San Jose, CA) and FlowJo Flow Cytometric Data Analysis Software (Tree Star, Inc. Ashland, OR).

### Cytotoxicity assay

A standard 4-hour chromium-release assay was used as previously described [26]. SKO-007(J3) cells stimulated as indicated above, were used as target cells and were labeled (100–200 μCi ^51^Cr/10^6^ cells; Amersham BioSciences, Piscataway, NJ) for 90 minutes at 37°C, washed, and 5 × 10^3^ cells/well were plated. As source of effector cells, we used primary NK cells as described above. The percentage of specific lysis was calculated by counting an aliquot of supernatant and using the formula: 100 × [(sample release - spontaneous release)/total release - spontaneous release)]. All determinations were made in triplicate, and E:T ratios ranged from 10:1 to 1:1, as indicated.

### Cell cycle analysis

SKO-007(J3) cell cycle distribution was analyzed by PI staining after 48 h drug treatment. Cells were washed in PBS with 0.1% sodium azide and fixed for 2 h at 4°C in cold 70% ethanol. Thereafter, cells were incubated for 30 min at room temperature with 50 μg/mL of PI in PBS containing 100 μg/mL of RNAse and immediately analyzed using a FACSCalibur flow cytometer. Flow cytometric analysis was performed using FlowJo software.

### Analysis of senescent cells

Senescence Associated *β*-galactosidase assay was performed using the fluorogenic substrate C12FDG to measure *β*-galactosidase activity by flow cytometry. Cells were incubated 1 h with 100 nM bafilomycin A1 to induce lysosomal alkalinization, followed by 1 h incubation with C12FDG (33 μM) and the C12-fluorescein signal of senescent cells was measured on the FL-1 detector using a FACSCalibur flow cytometer. Flow cytometric analysis was performed using FlowJo software.

### RNA isolation, RT-PCR and real-time PCR

Total RNA was extracted using TRIZOL™ (Life Technologies Inc., Grand Island, NY), according to manufacturer’s instructions. The concentration and quality of the extracted total RNA was determined by measuring light absorbance at 260 nm (A_260_) and the ratio of A_260_/A_280._ Reverse transcription was carried out in a 25 μl reaction volume with 2 μg of total RNA according to the manufacturer’s protocol for M-MLV reverse transcriptase (Promega, Madison, WI). Real-Time PCR was performed using the ABI Prism 7900 Sequence Detection system (Applied Biosystems, Foster City, CA). cDNAs were amplified in triplicate with primers for CD155/PVR (Hs00197846_m1) conjugated with fluorochrome FAM, and β-actin (4326315E) conjugated with fluorochrome VIC (Applied Biosystems). The level of ligand expression was measured using Ct (threshold cycle). The Ct was obtained by subtracting the Ct value of the gene of interest (PVR/CD155) from the housekeeping gene (β-actin) Ct value. In the present study we used Ct of the untreated sample as the calibrator. The fold change was calculated according to the formula 2^-ΔΔCt^, where ΔΔCt was the difference between Ct of the sample and the Ct of the calibrator (according to the formula, the value of the calibrator in each run is 1. The analysis was performed using the SDS version 2.2 software (Applied Biosystems, Foster City, CA).

### Western-blot analysis

For Western-Blot analysis, SKO-007(J3) cells were pelleted, washed once with cold phosphate-buffered saline, resuspended in lysis buffer [1% Nonidet P-40 (v/v), 10% glycerol, 0.1% SDS, 0.5% Sodium Deoxycholate, 1 mM phenyl-methyl-sulfonyl fluoride (PMSF), 10 mM NaF, 1 mM Na_3_VO_4_, COMPLETE protease1 inhibitor mixture (Roche, Indianapolis, IN) in PBS] and subsequently incubated 30 min on ice. The lysate was centrifuged at 14000 g for 15 min at 4°C and the supernatant was collected as whole cell extract. Protein concentration was determined by the BCA method (Pierce, Rockford, IL). Thirty to 50 μg of cell extract were run on 10% denaturing SDS-polyacrylamide gels. Proteins were then electroblotted onto nitrocellulose membranes (Schleicher & Schuell, Keene, NJ) and blocked in 3% milk in TBST buffer. Immunoreactive bands were visualized on the nitrocellulose membranes, using horseradish-peroxidase-coupled goat anti-rabbit or goat anti-mouse immunoglobulins and the ECL detection system (GE Healthcare Amersham), following the manufacturer’s instructions. Antibodies against phospho-Chk1 (Ser317), phospho-Chk2 (Thr68), total Chk1 and total Chk2 were purchased from Cell Signaling (Danvers, MA). Antibody against phospho-H2A.X was purchased from Millipore (Billerica, MA). Densitometric analysis was performed using Quantity One software (Bio-Rad, Hercules, CA).

## Results

### Nitric oxide upregulates expression of DNAM-1 ligand PVR/CD155 on human multiple myeloma cells

**Figure 1:**
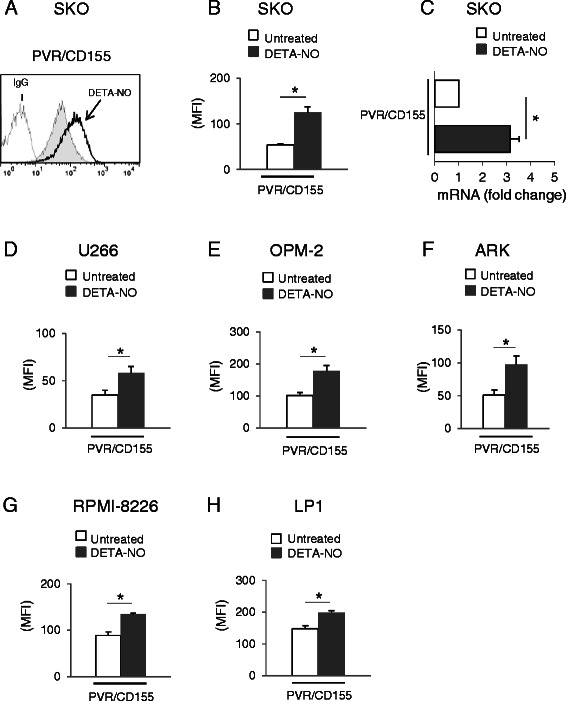
**Regulation of PVR/CD155 expression on MM cell lines following treatment with NO donor DETA-NO. A)** PVR/CD155 surface expression was analyzed by flow cytometry on SKO-007(J3) cells treated with DETA-NO (200 μM) for 48 h. Data are representative of one out of three independent experiments. The grey colored histogram represents basal expression, while thick black colored histogram represents the expression after treatment with DETA-NO. **B)** The MFI of PVR/CD155 surface expression was calculated based on at least four independent experiments and evaluated by paired Student *t* test (*P < 0.05). Histograms represent the MFI with specific mAb subtracted from the MFI value of isotype control. These treatments did not affect the cell viability over the time and DETA-NO concentration [200 μM for SKO-007(J3)] chosen for these experiments (as assessed by PI staining, data not shown). **C)** Real Time PCR analysis of total mRNA obtained from SKO-007(J3) cells, untreated (−) or treated with 200 μM DETA-NO for 24 h as described above. Data, expressed as fold change units, were normalized with β-actin and referred to the untreated cells considered as calibrator and represent the mean of 3 experiments (*P < 0.05). **D-H)** The MFI of PVR/CD155 surface expression was calculated for U266, OPM-2, ARK, RPMI-8226 and LP1 MM cells, based on at least three independent experiments and evaluated by paired Student *t* test (*P < 0.05). Histograms represent the MFI with specific mAb subtracted from the MFI value of isotype control. These treatments did not affect the cell viability over the time and DETA-NO concentration [200 μM for U266, 50 μM for OPM-2, 200 μM for ARK, 100 μM for RPMI-8226 and 125 μM for LP1] chosen for these experiments (as assessed by PI staining, data not shown).

In order to characterize novel agents and molecular pathways able to regulate the expression of NK cell activating ligands in MM cells, we investigated the activity of nitric oxide donors [DETA-NO and the NO-releasing pro-drugs NCX4040 (NO-aspirin) and JS-K] on the expression of the CD155/PVR, an activating DNAM-1 ligand regulated by DDR and reactive radicals in different models [23,24]. We initially performed a flow cytometric analysis on SKO-007(J3) MM cells after 48 h-treatment with DETA-NO, a donor able to release 2 moles of NO**•** per mole of compound and a half-life of 20 h at 37°C, ideal for the treatment of cells over long periods of time (e.g. 24–48 h). As shown in Figure 1A and B treatment of SKO-007(J3) cells upregulated basal surface expression of PVR/CD155 ligand; the concentration of DETA-NO used in these experiments (200 μM) has been chosen on the basis of dose–response assays using minimal doses of the donor [not affecting cell viability as assessed by PI staining (data not shown)], able to increase intracellular levels of NO• and to induce optimal PVR/CD155 expression (Additional file 1A and D). At this regard, 200 μM is within a concentration range of 0.1 to 1 mM DETA-NO, already shown to be equivalent to about 200 to 400 nM NO concentrations over a 24/48-hour period and comparable with reported NO concentrations at different sites of active inflammation [27,28].

Previous observations have shown that this cell line does not express detectable levels of the DNAM-1 ligand Nec-2/CD112, as well as the other cell lines used in this work (Additional file 2), and this ligand was not further analyzed in this study [21]. We next examined whether a possible mechanism underlying PVR/CD155 up-regulation on MM cells could be the consequence of an increased mRNA expression of this gene. Total RNA was isolated from SKO-007(J3) cells exposed to DETA-NO for 24 h and analyzed by Real-Time qRT-PCR. As shown in Figure 1C, we found a significant increase of PVR/CD155 mRNA levels in treated cells. We also investigated the effect of DETA-NO on other MM cell lines (U266, OPM-2, ARK, RPMI-8226 and LP-1) and confirmed that PVR/CD155 was similarly upregulated in all cell lines tested (Figure 1D-H). The concentration of DETA-NO used for the different cell lines has been chosen on the basis of dose–response assays using minimal doses of the donor not affecting cell viability and able to induce optimal PVR/CD155 expression (data not shown).

These results indicate that NO released by DETA-NO can enhance cell surface expression and mRNA levels of the DNAM-1 ligand PVR/CD155 in human MM cells.

### Exposure to nitric oxide increases degranulation and NK cell-mediated killing of MM cells

**Figure 2:**
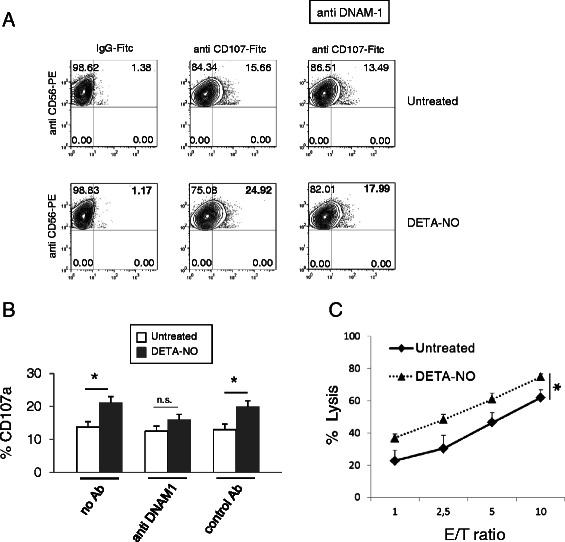
**NO exposed SKO-007(J3) cells enhances NK cell-mediated cytotoxicity. A)** NK cells prepared from PBMCs of healthy donors, were incubated with SKO-007(J3) cells, untreated or treated with DETA-NO for 48 h, and used as target cells in a degranulation assay. The assay was performed at the effector:target (E:T) ratio of 2.5:1. After 2 hours at 37°C, cells were stained with anti-CD56, anti-CD3 and anti-CD107a mAbs. Cell surface expression of CD107a was analyzed on CD56^+^CD3^−^ cells. In order to evaluate the role of DNAM-1, the assay was performed in parallel treating NK cells with blocking anti-DNAM-1 antibody. Results are representative of one out of three independent experiments. **B)** The MFI of CD107 were calculated based on at least three independent experiments and evaluated by paired Student *t* test (*P < 0.05). Histograms represent the MFI with specific mAb subtracted from the MFI value of isotype control. **C)** NK cells isolated from PBMCs of healthy donors were incubated with SKO-007(J3) cells, untreated or treated with DETA-NO for 48 h as described above, and used as target cells in a standard 4-hour chromium-release assay. The percentage of specific lysis was calculated by counting an aliquot of supernatant and using the formula: 100 x [(sample release - spontaneous release)/total release - spontaneous release)]. All determinations were made in triplicate and E:T ratios ranged from 10:1 to 1:1, as indicated. Data represent the mean (n = 3 experiments, *P < 0.05).

We tested whether treatment of myeloma cells with DETA-NO could lead to increased activation and NK cell-mediated killing. To this aim, we analyzed the degranulation activity of NK cells derived from healthy donors against SKO-007(J3) cells, evaluating the expression of the CD107a (a surrogate marker for granule mobilization) by FACS analysis. As shown in Figure 2A and B, basal expression of CD107a on NK cells contacting SKO-007(J3) was enhanced following treatment with DETA-NO. This increased degranulation was partially dependent on DNAM-1 activation, because significantly reduced in the presence of a blocking anti-DNAM-1 mAb. We also analyzed the possible role of the receptor TIGIT (T cell Ig and ITIM domain), a coinhibitory receptor that also binds to PVR/CD155 and Nec-2 ligands, expressed in NK cells as well as in different T cell subsets [29,30]; as shown in Additional file 3, the presence of a blocking anti-TIGIT mAb did not significantly modify basal or the increased degranulation induced by DETA-NO, suggesting that triggering of this receptor is not able to modulate the activity of NK cells, at least in this experimental setting. As a control for a possible direct effect of NO on NK cell functions, we also analyzed the degranulation activity of NK cells contacting SKO-007(J3) cells in the presence of DETA-NO; as shown in Additional file 4, degranulation activity was not significantly affected by the presence of the donor.

Finally, we analyzed the effect of DETA-NO on NK cell cytolytic function; as shown in Figure 2C, standard cytotoxicity assays using ^51^Cr-labeled SKO-007(J3) target cells were performed, and treatment with DETA-NO significantly increased specific killing when compared to the cytotoxicity of untreated cells.

Our results, therefore, indicate that increased expression of PVR/CD155 in SKO-007(J3) cells treated with DETA-NO enhances NK cell degranulation and killing by promoting DNAM-1 recognition.

### Molecular mechanisms involved in PVR/CD155 up-regulation by NO

**Figure 3:**
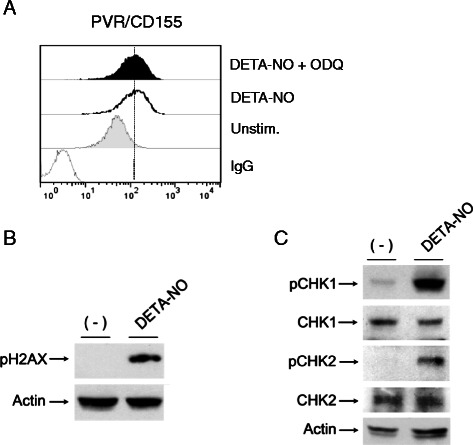
**NO enhances PVR/CD155 expression: molecular mechanisms. A)** PVR/CD155 surface expression was analyzed by flow cytometry on SKO-007(J3) cells treated with DETA-NO (200 μM) in the presence or absence of the guanylate cyclase inhibitor ODQ (50 μM) for 48 h. Data are representative of one out of three independent experiments. **B)** Western Blot analysis of total cellular proteins from SKO-007(J3) cells treated with DETA-NO for 18 h. The arrow indicates the expression of the pH2A.X and β-actin, used as loading control. The proteins transferred to nitrocellulose membranes were stained with Ponceau to verify that similar amounts of protein had been loaded in each lane. Data shown are representative of 1 out of 2 independent experiments. **C)** Western Blot analysis of total cellular proteins from SKO-007(J3) cells treated with DETA-NO for 18 h. Lysates were probed with antibodies to different phosphorylation sites of Chk1 and Chk2, wt Chk1 and Chk2 or β-actin, used as loading control. The proteins transferred to nitrocellulose membranes were stained with Ponceau to verify that similar amounts of protein had been loaded in each lane. Data shown are representative of 1 out of 2 independent experiments.

One of the most studied mechanisms involved in physiological pathways regulated by NO is the activation of the heme iron in the soluble guanylate cyclase (sGC), able to stimulate cGMP production and activation of downstream signalling [31,32]. To determine whether this molecular pathway might be involved in PVR/CD155 up-regulation in MM cells, SKO-007(J3) cells were treated with DETA-NO in the presence or absence of ODQ, a widely used specific inhibitor of soluble guanylate cyclase used to differentiate cGMP-mediated effects of NO from cGMP-independent effects [33,34]. However, as shown in Figure 3A, up-regulation of PVR/CD155 was not affected by ODQ, suggesting that cGMP-mediated signalling was not involved.

**Figure 4:**
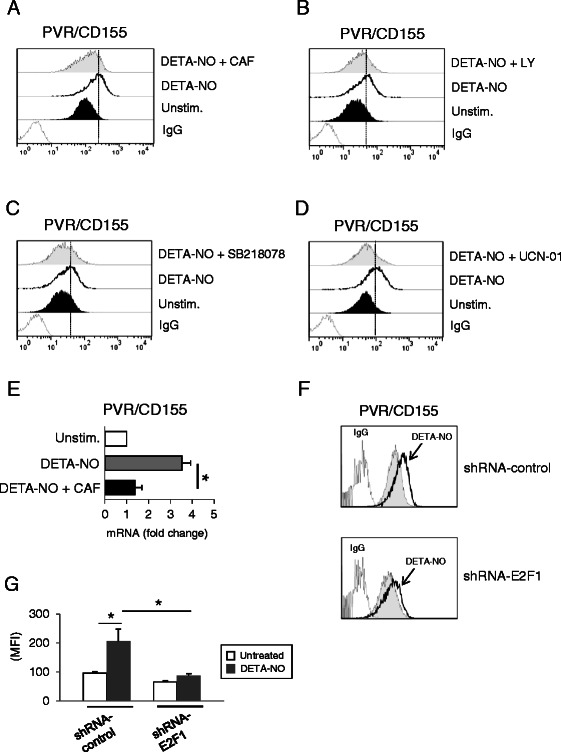
**NO enhances PVR/CD155 expression: role of DDR. A,B)** PVR/CD155 surface expression was analyzed by flow cytometry on SKO-007(J3) cells treated with DETA-NO (200 μM) in the presence or absence of caffeine (CAF 1 mM) or LY294002 (LY 20 μM) for 48 h. Data are representative of one out of four independent experiments. **C,D)** PVR/CD155 surface expression was analyzed by flow cytometry on SKO-007(J3) cells treated with DETA-NO (200 μM) in the presence or absence of the Chk1/2 inhibitors SB218078 and UCN-01 (0.5 μM and 50 nM respectively) for 48 h. Data are representative of one out of four independent experiments. In these experiments, the concentration used for the different inhibitors, did not significantly affect cell viability as assessed by PI staining (data not shown). **E)** Real Time PCR analysis of total mRNA obtained from SKO-007(J3) cells, treated for 24 h in the presence or absence of caffeine (1 mM) as described above. Data, expressed as fold change units, were normalized with β-actin and referred to the untreated cells considered as calibrator and represent the mean of 3 experiments (*P < 0.05). **F)** PVR/CD155 surface expression was analyzed by flow cytometry on SKO-007(J3) non-target shRNA (shRNA-control) or pLKO-sh-E2F1 cells, treated with DETA-NO as described above. Data are representative of one out of three independent experiments. **G)** The MFI of PVR/CD155 surface expression was calculated based on at least three independent experiments and evaluated by paired Student *t* test (*P < 0.05). Histograms represent the MFI with specific mAb subtracted from the MFI value of isotype control.

Nitric oxide can also interact directly with biological target molecules, nonetheless, when generated in high amounts such as during inflammation, it can exert indirect effects, reacting with superoxide anion to produce different reactive nitrogen species (RNS) [e.g. peroxynitrite (a strong oxidant)] with significant pathophysiological/inflammatory actions (reviewed in [35,36]). In this regard, the different actions of NO in tumor biology may be in part explained by the complex dose-dependent interactions of NO and the related RNS with DNA, producing both single and double-strand breaks and genotoxic stress [20,37]. As our laboratory has recently shown that genotoxic drugs (e.g. melphalan or doxorubicin) can trigger the expression of NK activating ligands on MM cells in an ATM/ATR/Chk1/2-dependent and p53-independent manner [12,24], we investigated the possibility that a similar mechanism might be involved in the presence of NO donors. We analyzed the activation of ATM/ATR-dependent down-stream signalling components, such as H2A.X and Chk1/2 kinases, already described to phosphorylate and activate effector proteins that inhibit cell cycle progression and to activate DNA repair [38,39]; as shown in Figure 3B and C, DETA-NO was able to induce H2A.X phosphorylation on residue Ser139 (pH2A.X) and Chk1 and Chk2 phosphorylation on Ser317 and Thr68, respectively. In this regard, as shown in Figure 4A and B (and Additional file 5A,B), up-regulation of PVR/CD155 expression was significantly inhibited by caffeine or by LY294002 in SKO-007(J3) cells, two widely used inhibitors capable of blocking both ATM and ATR catalytic activity [40], and by SB218078 or UCN-01, inhibitors of Chk1/2 kinases (Figure 4C,D and Additional file 5C,D). Accordingly, we also found a significant inhibition of PVR/CD155 mRNA levels in DETA-NO + caffeine-treated cells (Figure 4E) and, in addition, up-regulation of PVR/CD155 expression was significantly inhibited in SKO-007(J3) cells in which the expression of E2F1 was reduced by shRNA interference (already described in [24]), a transcription factor activated/stabilized by ATM/ATR and Chk2 [41-43] and recently shown to upregulate the expression of PVR/CD155 in MM cells exposed to genotoxic drugs [24]. These results indicate that NO-mediated activation of DDR is involved in the up-regulation of PVR/CD155 in MM cells.

### NO/DDR-induced up-regulation of PVR/CD155 is not related to a senescence-dependent mechanism

**Figure 5:**
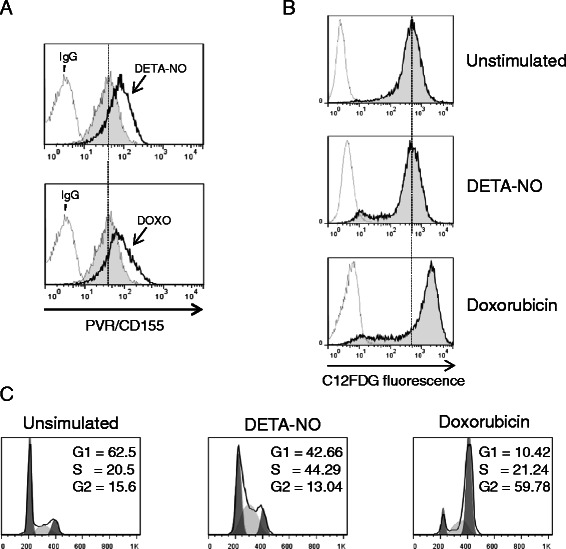
**NO-induced up-regulation of PVR/CD155 is not related to a senescence-dependent mechanism. A)** PVR/CD155 surface expression was analyzed by flow cytometry on SKO-007(J3) cells treated with DETA-NO (200 μM) or with doxorubicin (0.05 μM) for 48 h. Data are representative of one out of three independent experiments. The grey colored histograms represent basal expression of the indicated ligand, while thick black colored histograms represent the expression after treatment with the indicated drug. **B)** SA-*β*Gal activity of SKO-007(J3) cells treated with DETA-NO or with doxorubicin for 48 h as described above. Data are representative of one out of three independent experiments. The grey colored histograms represent the C12-fluorescein signal. **C)** SKO-007(J3) cells were treated for 48 hours with the indicated drug as described above. Cells were fixed and stained with PI to analyze cell distribution among the different cell-cycle phases.

We have previously demonstrated that genotoxic drugs (e.g. doxorubicin)-induced up-regulation of PVR/CD155 is associated with a senescence-dependent G2/M cell cycle arrest in MM cells [12]. Here, we investigated the possible link between DDR, cell cycle, induction of senescence and the ability of NO to induce PVR/CD155 expression. As shown in Figure 5A, stimulation of SKO-007(J3) cells with DETA-NO or with doxorubicin increased basal cell surface expression of PVR/CD155; however, only doxorubicin could activate a senescence-dependent G2/M cell cycle arrest (Figure 5B and C) as indicated by the different levels of SA-*β*Gal activity and G2/M quantification. These data suggest that different (DDR)-related pathways may be triggered by these drugs and that cellular senescence is not correlated or involved in NO-induced up-regulation of PVR/CD155 in MM cells.

### Anticancer nitric oxide-releasing prodrugs upregulates expression of PVR/CD155 on human multiple myeloma cells

Rational design of pharmacological agents (including NO-donors) takes account of specific modifications of known molecules with the purpose of optimizing their properties mainly in terms of efficacy and safety. In this context, the use of compounds that generate NO spontaneously for the treatment of malignancies is precluded due to the potential general toxic effects of NO. Thus, we investigated also the activity of novel prototype anticancer NO-releasing prodrugs on the expression of PVR/CD155. To this aim, we treated SKO-007(J3) cells with the NO-releasing aspirin derivative NCX4040 (a bio-activated nitric oxide-donating non-steroidal anti-inflammatory drug) [44] or with JS-K, an anti-cancer agent designed to release nitric oxide in a sustained manner within a cell when metabolized by glutathione *S*-transferases (GSTs), enzymes frequently overexpressed in different tumors, including MM [45,46]. The concentration of donors used in these experiments have been chosen on the basis of dose–response assays using minimal doses of the specific donor (not affecting cell viability as assessed by PI staining, data not shown), able to induce optimal PVR/CD155 expression (Additional file 1B and C).

**Figure 6:**
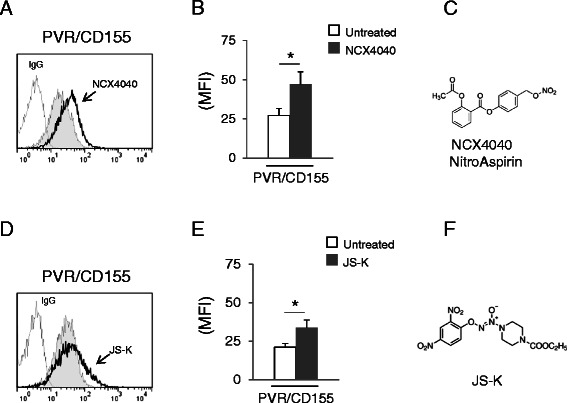
**Regulation of PVR/CD155 expression on MM cell lines following treatment with the NO-releasing prodrugs NitroAspirin and JS-K. A)** PVR/CD155 surface expression was analyzed by flow cytometry on SKO-007(J3) cells treated with NCX4040 (10 μM) for 48 h. Data are representative of one out of three independent experiments. The grey colored histogram represents basal expression of the indicated ligand, while thick black colored histogram represents the expression after treatment with NCX4040. **B)** The MFI of PVR/CD155 surface expression was calculated based on at least three independent experiments and evaluated by paired Student *t* test (*P < 0.05). Histograms represent the MFI with specific mAb subtracted from the MFI value of isotype control. **C)** Molecular structure of NCX4040. **D)** PVR/CD155 surface expression was analyzed by flow cytometry on SKO-007(J3) cells treated with JS-K (3 μM) for 48 h. Data are representative of one out of three independent experiments. The grey colored histogram represents basal expression of the indicated ligand, while thick black colored histogram represents the expression after treatment with JS-K. **E)** The MFI of PVR/CD155 surface expression was calculated based on at least three independent experiments and evaluated by paired Student *t* test (*P < 0.05). Histogram represents the MFI with specific mAb subtracted from the MFI value of isotype control. **F)** Molecular structure of JS-K. The concentration of the indicated donor used in these experiments, did not significantly affect cell viability as assessed by PI staining (data not shown).

As shown in Figure 6, treatment of SKO-007(J3) cells with NCX4040 or with JS-K at micromolar concentrations (known to generate significant levels of intracellular NO in different cell lines, including MM [44-46]), upregulated the basal cell surface expression of PVR/CD155, confirming the data obtained using DETA-NO and suggesting the use of novel NO-releasing prodrugs as an additional class of regulators of the expression of DNAM-1 ligand in cancer cells.

## Discussion and conclusion

Anticancer immune responses may contribute to the control of tumors after conventional chemotherapy and different observations have indicated that chemotherapeutic agents (e.g. genotoxic drugs) or adjuvant radiotherapy can induce immune responses that result in immunogenic cancer cell death or immunostimulatory side effects [47-50]. In this regard, increasing experimental and clinical evidence highlight the importance of NK cells in immune responses toward MM and combination therapies able to enhance the activity of NK cells against MM are showing promise in treating this hematologic cancer. Recently, a novel connection between therapeutic immuno-modulation and chemotherapy has been the finding that anti-cancer drugs (e.g. genotoxic agents, inhibitors of histone deacetylases, of the proteasome or of the HSP-90 chaperone) can increase the expression of DNAM-1 and NKG2D activating ligands, thus enhancing the response of receptor-expressing lymphocytes (NK cells, NKT cells and CTLs) against tumor cells, including MM [11,12,21,24,51-54].

Different and contradictory results have been reported about the role of nitric oxide in cancer progression, metastases and treatment of disease (reviewed in [19,20]). Initial findings suggested that immune cell-generated NO can be cytostatic or cytotoxic for a number of tumors; indeed, several reports have shown that macrophages can selectively destroy different tumor types (*in vitro* and *in vivo*) through the production of high levels of NO [55-58]. Moreover, NO can also enhance the cytotoxicity of NK cells and regulate survival of dendritic cells [59-61] and its release in models of lung and hepatic metastases microvasculature has been associated to a natural local defense mechanism inducing tumor cell killing [62,63]. On the other hand, other findings highlighted opposite actions mediated by NO, leading to increased tumor growth; in this context, low concentrations of NO have been shown to promote invasion and metastases (reviewed in [17,20]) and production of NO within specific tumor microenvironments has been described to enhance tumor progression, mainly by stimulating angiogenesis and/or to repress T cell responses by CD11b^+^/Gr-1^+^ myeloid cells (reviewed in [17]).

The observations described in this work can provide additional information on the role of nitric oxide in cancer and in MM. In particular, we investigated the effect of nitric oxide on the expression of the DNAM-1 ligand PVR/CD155 in MM cells. We found that treatment of MM cell lines with nitric oxide donors (DETA-NO, NitroAspirin/NCX4040 or JS-K) can increase the expression of this ligand, rendering these cells more susceptible to NK cell-mediated killing (Figure 2). Moreover, we identified one of the possible mechanism(s) involved in this up-regulation, the activation of a DNA damage response, a molecular pathway already described to regulate the expression of NK cells activating ligands in several cellular models [12,24,64]. NO-generated nitrogen species [20,37] and the consequent production of single and/or double DNA strand breaks can activate DDR in MM cells (as shown in Figure 3B and C); in this regard, upregulation of PVR/CD155 by DETA-NO was significantly reduced by inhibitors of ATM/ATR catalytic activity (caffeine and LY294002) and by inhibitors of the Chk1/2 kinases (SB218078 and UCN-01) (Figure 4C-D). In addition, silencing of E2F1, a transcription factor activated/stabilized by ATM/ATR/Chk2 [41-43] and described to upregulate the expression of PVR/CD155 in MM cells exposed to genotoxic drugs [24], resulted in a marked reduction of PVR/CD155 up-regulation (Figure 4E and F). These results indicate that NO-mediated activation of DDR is involved in the up-regulation of PVR/CD155 and that one of the mechanism(s) underlying this regulation implicates the activity of E2F1. Interestingly, and differently from our previous observation that up-regulation of PVR/CD155 is preferentially associated with a senescence-dependent G2/M cell cycle arrest [12], NO failed to activate a senescence and G2/M cell cycle arrest in our experimental system, as indicated by the different levels of SA-*β*Gal activity and G2/M phase between DETA-NO and doxorubicin-treated cells (used here as positive control) (Figure 5B and C). These data suggest that specific molecular pathways activated by RNSs and/or a different strength of DDR might be induced by these drugs and that cellular senescence is not correlated or involved in up-regulation of PVR/CD155. Moreover, the three NO-donors used in this work differ in their capability to upregulate PVR/CD155 expression, at least in our experimental setting of donor concentration and duration of treatment (as shown in Figures 1 and 6); these differences might reflect the possibility that additional molecular action(s) besides NO release might contribute to donors biologic activities, in particular mediated by the aspirin-moiety (NCX4040) or by the JS-K’s arylating ability on different nucleophilic biomolecules [65]. Further experiments will be needed to better characterize possible differences in activation of DDR by these drugs and the correlation with the expression of activating ligands.

Work by other groups has demonstrated a direct cytotoxic/anti-myeloma activity of NO as a consequence of induction of DDR, using the NO-releasing prodrug JS-K [46], which can also affect the interaction of MM cells with bone marrow microenvironment, modulating tumor angiogenesis *in vivo* and *in vitro* [66]. Moreover, NO can function as a negative feedback signal to limit pathologic osteoclastogenesis via RANKL/iNOS/NO autoregulatory pathway [67]. In a different context, treatment with JS-K or the activation of macrophage-dependent NO expression after IL-2 + anti-CD40 immunotherapy has been shown to modulate metastatic progression in an orthotopic model of renal cell carcinoma [68]. Similarly, local production of significant amounts of NO by iNOS^+^ has been also shown to deeply affect the activity of pro-tumoral microenvironments, as demonstrated using neoadjuvant local low-doses of gamma irradiation (LDI) in a model of pancreatic carcinogenesis [69]; in this model, LDI is able to redirect local (or pre-adoptive-transfer) macrophage differentiation from a cancer-promoting immunosuppressive state to an iNOS^+^ phenotype, to normalize aberrant angiogenesis-driven vascular abnormalities and to enable infiltration of cytotoxic T cells. In this regard, local MM-associated macrophages play a crucial role in the pathophysiology of MM and can promote plasma cell growth with aberrant vasculogenesis (reviewed in [70]); moreover, hypoxia-mediated impairment of NO signalling can also contribute to tumor escape from NK cell immunesurveillance by inducing shedding of the NKG2DL MICA, through a mechanism involving increased expression/activity of ADAM10 via HIF-1α [71,72].

The possibility to regulate activating ligands such as PVR/CD155 in MM cells, able to enhance the activity of cytotoxic lymphocytes (e.g. NK cells) by pharmacological delivery of NO-releasing prodrugs (also in combined immunotherapy) or local production of NO by “therapy-reprogrammed” or adoptively transferred iNOS^+^ macrophages, might be considered as an additional strategy to hit the tumor and to modify local microenvironment allowing and/or enhancing immuno-therapeutic applications.

## Additional files

Additional file 1: **A-C) Dose–response assays using minimal doses of the indicated donor (not affecting cell viability as assessed by PI staining, data not shown) able to induce optimal PVR/CD155 expression in SKO-007(J3) cells after 48 h treatment.** The optimal doses chosen (indicated in bold) were: DETA-NO 200 μM, NCX4040 10 μM, JS-K 3 μM. D) Intracellular NO**•** levels in SKO-007(J3) cells after 24 h treatment with DETA-NO 200 μM.
Additional file 2: **Nec-2/CD112 surface expression was analyzed by flow cytometry on SKO-007(J3), U266, OPM-2, ARK, RPMI-8226 and LP1 MM cells.** K562 cells were used here as positive control. The thin black colored histogram represents IgG-control while grey colored histogram represents the expression of Nec-2.
Additional file 3: **NK cells prepared from PBMCs of healthy donors were incubated with SKO-007(J3) cells, untreated or treated with DETA-NO for 48 h, and used as target cells in a degranulation assay.** The assay was performed at the effector:target (E:T) ratio of 2.5:1. After 2 hours at 37°C, cells were stained with anti-CD56, anti-CD3 and anti-CD107a mAbs. Cell surface expression of CD107a was analyzed on CD56^+^CD3^−^ cells. In order to evaluate the role of TIGIT, the assay was performed in parallel treating NK cells with blocking anti-TIGIT antibody. Results are representative of one out of two independent experiments.
Additional file 4: **NK cells prepared from PBMCs of healthy donors were incubated with SKO-007(J3) cells as described above, and used as target cells in a degranulation assay.** The assay was performed at the effector:target (E:T) ratio of 2.5:1, in the presence or in the absence of DETA-NO 200 μM. After 2 hours at 37°C, cells were stained with anti-CD56, anti-CD3 and anti-CD107a mAbs. Cell surface expression of CD107a was analyzed on CD56^+^CD3^−^ cells. Results are representative of one out of two independent experiments.
Additional file 5: **A,B) PVR/CD155 surface expression was analyzed by flow cytometry on SKO-007(J3) cells treated with DETA-NO (200 μM) in the presence or absence of caffeine (CAF 1 mM) or LY294002 (LY 20 μM) for 48 h.** C,D) PVR/CD155 surface expression was analyzed by flow cytometry on SKO-007(J3) cells treated with DETA-NO (200 μM) in the presence or absence of the Chk1/2 inhibitors SB218078 and UCN-01 (0.5 μM and 50 nM respectively) for 48 h. The MFI of PVD/CD155 expression was calculated based on at least four independent experiments and evaluated by paired Student *t* test (**P* < 0.05). Histograms represent the MFI with specific mAb subtracted from the MFI value of isotype control.

## Abbreviations

DDR: DNA Damage Response
DNAM-1: DNAX accessory molecule-1
GSTs: Glutathione *S*-transferases
MM: Multiple Myeloma
NCR: Natural Cytotoxicity Receptors
Nec-2: Nectin-2-CD112
NKG2D: NK group 2D
PVR: Poliovirus receptor-CD155
RNS: Reactive nitrogen species
ROS: Reactive oxygen species
TIGIT: T cell Ig and ITIM domain
