# Supplementary material for: Nitric oxide donors increase PVR/CD155 DNAM-1 ligand expression in multiple myeloma cells: role of DNA damage response activation
Source: BMC Cancer. 2015 Jan 22;15:17. doi: 10.1186/s12885-015-1023-5 (PMC4311457; doi:10.1186/s12885-015-1023-5)
Supplement: Additional file 1: — A-C) Dose–response assays using minimal doses of the indicated donor (not affecting cell viability as assessed by PI staining, data not shown) able to induce optimal PVR/CD155 expression in SKO-007(J3) cells after 48 h treatment. The optimal doses chosen (indicated in bold) were: DETA-NO 200 μM, NCX4040 10 μM, JS-K 3 μM. D) Intracellular NO• levels in SKO-007(J3) cells after 24 h treatment with DETA-NO 200 μM. [file 12885_2015_1023_MOESM1_ESM.pptx]

## Slide 1
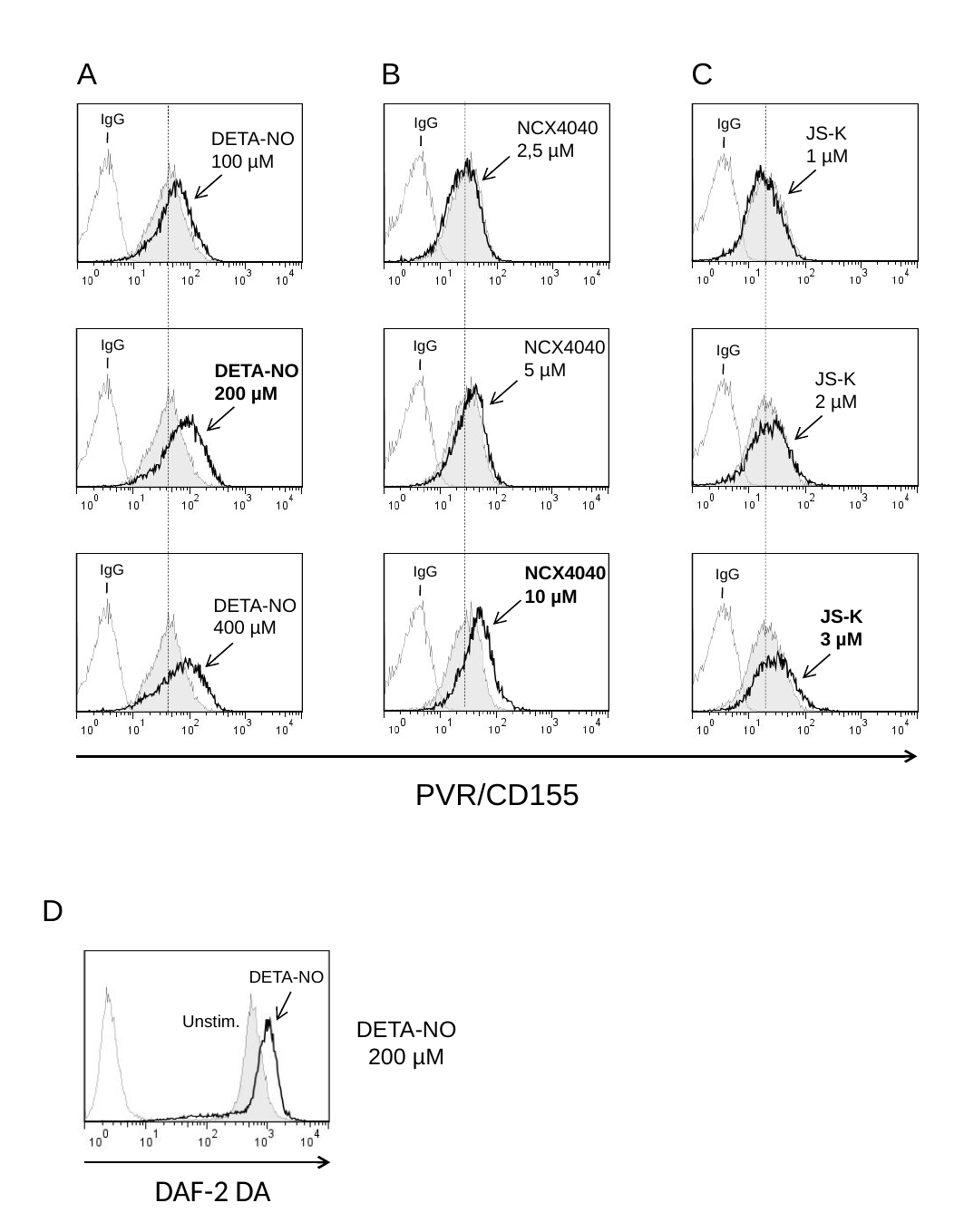

A
B
C
IgG
IgG
IgG
NCX4040
2,5 µM
JS-K
1 µM
DETA-NO
100 µM
IgG
NCX4040
5 µM
IgG
IgG
DETA-NO
200 µM
JS-K
2 µM
IgG
NCX4040
10 µM
IgG
IgG
DETA-NO
400 µM
JS-K
3 µM
PVR/CD155
D
DETA-NO
Unstim.
DETA-NO
200 µM
DAF-2 DA
