# Supplementary figures and images for: Nitric oxide donors increase PVR/CD155 DNAM-1 ligand expression in multiple myeloma cells: role of DNA damage response activation
Source: BMC Cancer. 2015 Jan 22;15:17. doi: 10.1186/s12885-015-1023-5 (PMC4311457; doi:10.1186/s12885-015-1023-5)

## Slide 1
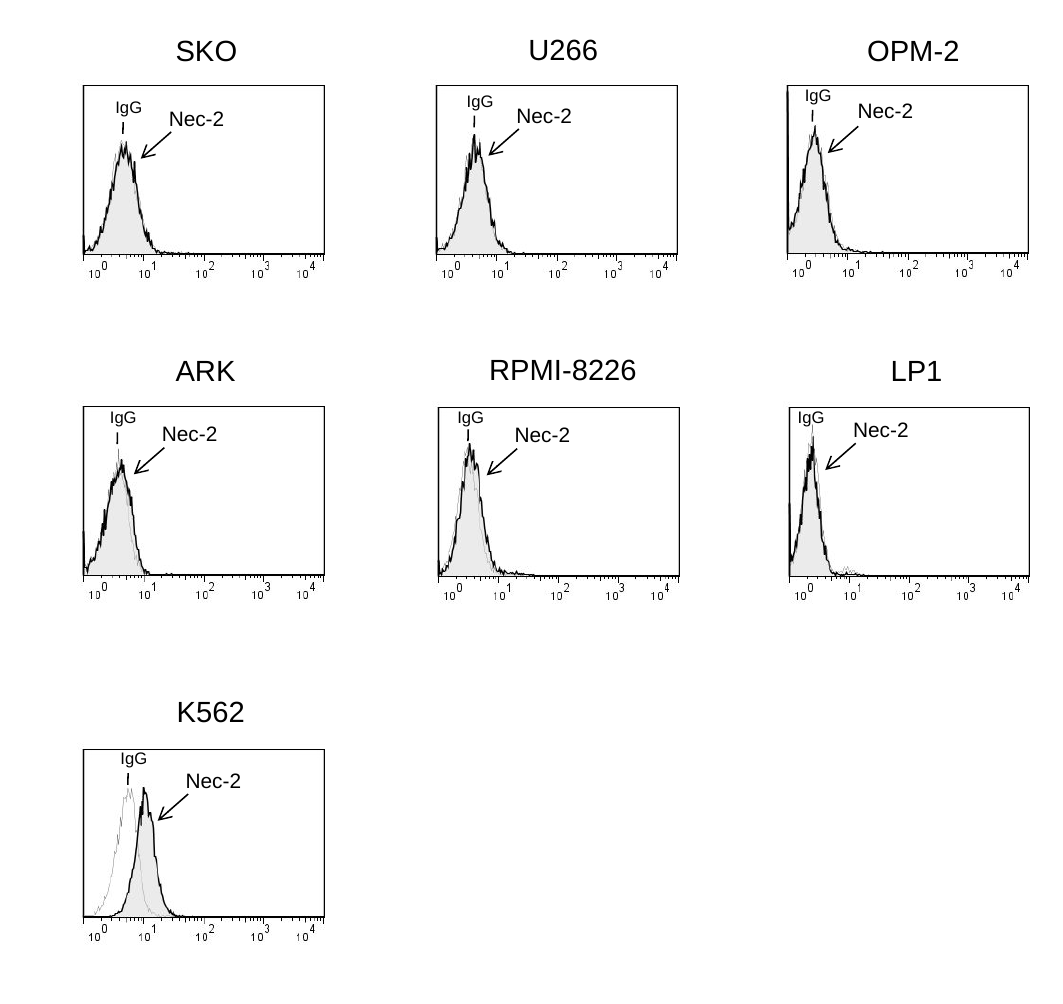

U266
SKO
OPM-2
IgG
IgG
IgG
Nec-2
Nec-2
Nec-2
RPMI-8226
ARK
LP1
IgG
IgG
IgG
Nec-2
Nec-2
Nec-2
K562
IgG
Nec-2

Supplement: Additional file 2: — Nec-2/CD112 surface expression was analyzed by flow cytometry on SKO-007(J3), U266, OPM-2, ARK, RPMI-8226 and LP1 MM cells. K562 cells were used here as positive control. The thin black colored histogram represents IgG-control while grey colored histogram represents the expression of Nec-2. [file 12885_2015_1023_MOESM2_ESM.pptx]

## Slide 1
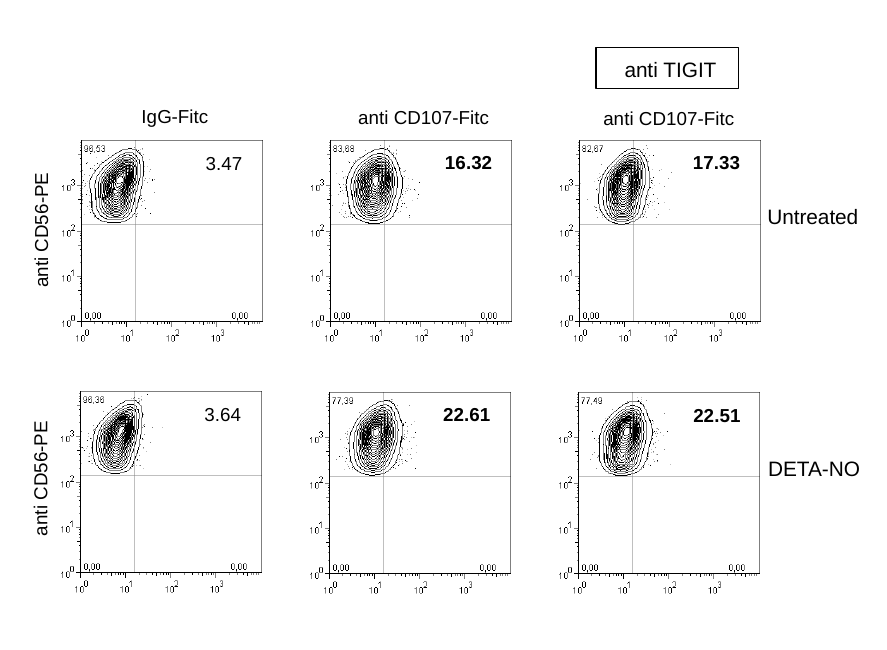

anti TIGIT
IgG-Fitc
anti CD107-Fitc
anti CD107-Fitc
17.33
16.32
3.47
Untreated
anti CD56-PE
3.64
22.61
22.51
DETA-NO
anti CD56-PE

Supplement: Additional file 3: — NK cells prepared from PBMCs of healthy donors were incubated with SKO-007(J3) cells, untreated or treated with DETA-NO for 48 h, and used as target cells in a degranulation assay. The assay was performed at the effector:target (E:T) ratio of 2.5:1. After 2 hours at 37°C, cells were stained with anti-CD56, anti-CD3 and anti-CD107a mAbs. Cell surface expression of CD107a was analyzed on CD56+CD3− cells. In order to evaluate the role of TIGIT, the assay was performed in parallel treating NK cells with blocking anti-TIGIT antibody. Results are representative of one out of two independent experiments. [file 12885_2015_1023_MOESM3_ESM.pptx]

## Slide 1
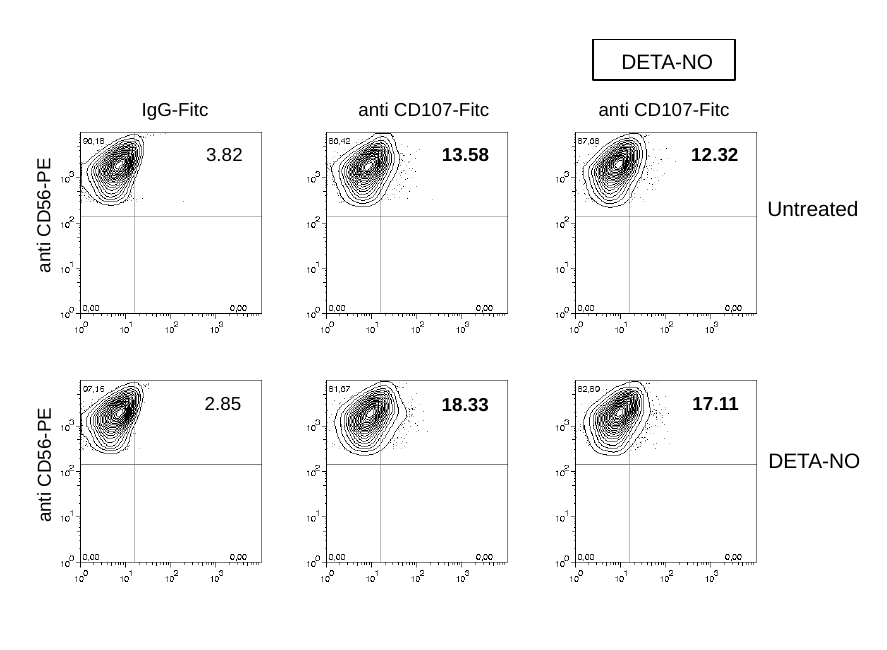

DETA-NO
IgG-Fitc
anti CD107-Fitc
anti CD107-Fitc
3.82
13.58
12.32
Untreated
anti CD56-PE
17.11
2.85
18.33
DETA-NO
anti CD56-PE

Supplement: Additional file 4: — NK cells prepared from PBMCs of healthy donors were incubated with SKO-007(J3) cells as described above, and used as target cells in a degranulation assay. The assay was performed at the effector:target (E:T) ratio of 2.5:1, in the presence or in the absence of DETA-NO 200 μM. After 2 hours at 37°C, cells were stained with anti-CD56, anti-CD3 and anti-CD107a mAbs. Cell surface expression of CD107a was analyzed on CD56+CD3− cells. Results are representative of one out of two independent experiments. [file 12885_2015_1023_MOESM4_ESM.pptx]
