# Supplementary material for: Nitric oxide donors increase PVR/CD155 DNAM-1 ligand expression in multiple myeloma cells: role of DNA damage response activation
Source: BMC Cancer. 2015 Jan 22;15:17. doi: 10.1186/s12885-015-1023-5 (PMC4311457; doi:10.1186/s12885-015-1023-5)
Supplement: Additional file 5: — A,B) PVR/CD155 surface expression was analyzed by flow cytometry on SKO-007(J3) cells treated with DETA-NO (200 μM) in the presence or absence of caffeine (CAF 1 mM) or LY294002 (LY 20 μM) for 48 h. C,D) PVR/CD155 surface expression was analyzed by flow cytometry on SKO-007(J3) cells treated with DETA-NO (200 μM) in the presence or absence of the Chk1/2 inhibitors SB218078 and UCN-01 (0.5 μM and 50 nM respectively) for 48 h. The MFI of PVD/CD155 expression was calculated based on at least four independent experiments and evaluated by paired Student t test (*P < 0.05). Histograms represent the MFI with specific mAb subtracted from the MFI value of isotype control. [file 12885_2015_1023_MOESM5_ESM.pptx]

## Slide 1
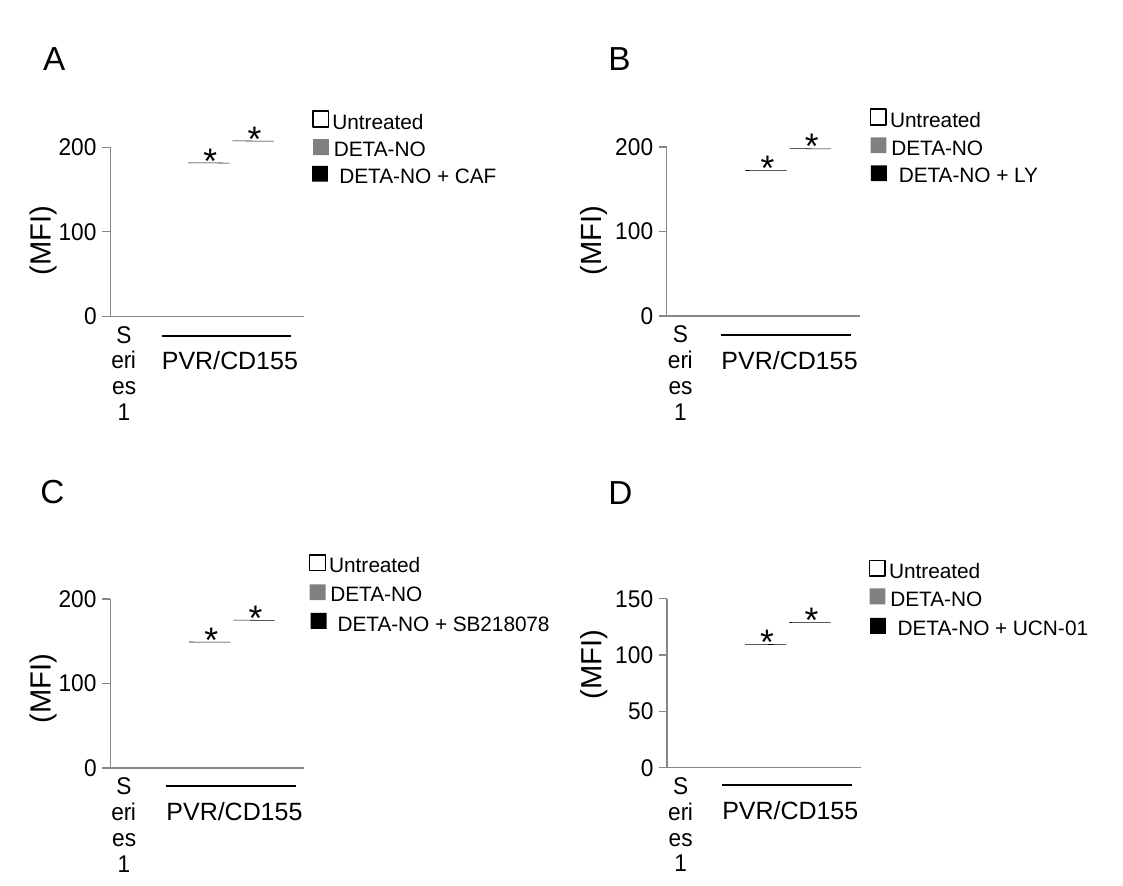

B
A
Untreated
Untreated
*
*
### Chart
| Category | |
|---|---|
| | None |
| | 76.9 |
| | 143.3 |
| | 80.96 |
| | None |
### Chart
| Category | |
|---|---|
| | None |
| | 68.19 |
| | 149.82 |
| | 99.0 |
| | None |DETA-NO
DETA-NO
*
*
DETA-NO + LY
DETA-NO + CAF
(MFI)
(MFI)
PVR/CD155
PVR/CD155
C
D
Untreated
Untreated
DETA-NO
### Chart
| Category | |
|---|---|
| | None |
| | 43.2 |
| | 90.0 |
| | 50.4 |
| | None |
### Chart
| Category | |
|---|---|
| | None |
| | 60.0 |
| | 123.86 |
| | 80.94 |
| | None |DETA-NO
*
*
DETA-NO + SB218078
DETA-NO + UCN-01
*
*
(MFI)
(MFI)
PVR/CD155
PVR/CD155
